# Betulin Suppresses Osteoclast Formation via Down-Regulating NFATc1

**DOI:** 10.3390/jcm7060154

**Published:** 2018-06-15

**Authors:** Kwang-Jin Kim, Yongjin Lee, Hae-Gwang Hwang, Sang Hyun Sung, Mina Lee, Young-Jin Son

**Affiliations:** 1 Department of Pharmacy, Sunchon National University, Jeonnam, Suncheon 57922, Korea; mastiffk@naver.com (K.-J.K.); yojilee@gmail.com (Y.L.); winhhk@naver.com (H.-G.H.); 2 College of Pharmacy and Research Institute of Pharmaceutical Sciences, Seoul National University, Gwanak-gu, Seoul 08826, Korea; shsung@snu.ac.kr

**Keywords:** bone, osteoporosis, osteoclast, RANKL, NFATc1, betulin, natural product, *Betula platyphylla*

## Abstract

Osteoporosis is a disease characterized by osteoclast-mediated low bone mass. The modulation of osteoclasts is important for the prevention or therapeutic treatment of loss of bone mass. Osteoclasts, which are bone-resorbing multinucleated cells, are differentiated from the hematopoietic stem cell monocyte/macrophage lineage by Receptor activator of nuclear factor kappa-B ligand (RANKL) expressed from osteoblasts and stromal cells. RANKL signaling ultimately activates nuclear factor of activated T Cells 1 (NFATc1), which is a master transcription factor in osteoclastogenesis. Betulin, a lupine type pentacyclic triterpenoid, was isolated from the bark of *Betula platyphylla*. Betulin inhibited RANKL-mediated osteoclast differentiation by downregulating NFATc1. Betulin may serve as a useful structural scaffold in the therapeutic agent development to prevention/treatment the osteoclast-mediated bone disorder.

## 1. Introduction

It is important to maintain the homeostasis of bone mass through the balance between the activities of osteoclasts and osteoblasts. The breaking of this homeostasis will lead to bone diseases, such as osteoporosis, rheumatoid arthritis, and multiple myeloma-related forms of bone loss [1,2]. Osteoclasts, which are bone resorbing cells, differentiate into tartrate-resistant acid phosphatase (TRAP)-positive cells, and fuse to multinucleated cells from a monocyte-macrophage lineage of hematopoietic stem cells; this process is important in bone remodeling [3,4]. Osteoclasts attach to a bone surface and secrete various types of acids and lytic enzymes, such as protons, TRAP, and cathepsin K for bone resorption [5,6].

Osteoclastogenesis is induced by receptor activator of nuclear factor kappa-B ligand (RANKL), also known as TNF Superfamily Member 11 (TNFSF11), osteoclast differentiation factor (ODF), and TNF-related activation-induced cytokine (TRANCE), in the presence of the macrophage colony-stimulating factor (M-CSF) [7,8,9]. RANKL, expressed by osteoblasts/stromal cells, belongs to the tumor necrosis factor (TNF) superfamily and binds to the RANK on the surface of osteoclast precursors [10]. In particular, the RANKL-RANK signaling pathway plays an important role in osteoclast differentiation through the expression of the NFATc1, which modulates osteoclast-specific genes, such as TRAP, dendritic cell-specific transmembrane protein (DC-STAMP), calcitonin receptor, osteoclast-associated receptor (OSCAR), and cathepsin K [11,12,13,14]. 

Historically, natural products are important resources in drug discovery and have been developed for a variety of medicines, such as immunosuppressive agents, anticancer agents, and enzyme inhibitors [15,16]. There have been recent efforts to find drugs from natural products that prevent and treat bone diseases while minimizing adverse side effects [17,18]. Recently, we isolated betulin from the inner bark of *Betula platyphylla* with chloroform, butanol, and water. It is a lupane type pentacyclic triterpenoid, commonly isolated from the bark of yellow and white birch trees [19], and has pharmacological properties, such as anti-tumor activity, anti-inflammatory activity, anti-viral activity, and antibacterial activity [20,21]. Also, Betulin can be derived from Betulinic acid, which has been reported to suppress osteoclast differentiation and bone resorption. However, the effect of Betulin has not yet been reported [22,23]. Therefore, we investigated the effects of betulin on osteoclastogenesis and analyzed its molecular mechanisms in this report.

## 2. Materials and Methods

### 2.1. Plant Material

*B*. *platyphylla* inner bark was provided by SK E&C (Chungju, Korea) and collected in the afforested land of SK E&C, which has more than 450,000 *B*. *platyphylla* in 167.6 ha of forested area in Chungju, Korea. A voucher specimen (SNU-797) has been deposited in the herbarium of the medicinal plant garden, College of Pharmacy, Seoul National University, Koyang, Korea.

### 2.2. Extraction and Isolation

Dried and pulverized *B*. *platyphylla* bark (5.7 kg) was extracted with 80% methanol (30 L, 3 h × 4) by ultrasonication at room temperature, and was concentrated in vacuo to afford a crude extract (984.4 g), which was suspended in H_2_O and partitioned in CHCl_3_ (205.3 g), *n*-butanol (577.5 g), and H_2_O (160.5 g) fractions. Betulin was produced by recrystallization from *n*-butanol and H_2_O fraction of *B*. *platyphylla* bark. ^1^H- and ^13^C-NMR spectra of betulin were obtained on a JEOL 400 NMR spectrometer (400 and 100 MHz for 1H and 13C, respectively; Tokyo, Japan) in CDCl3 with solvent signals as internal standards. The purity of betulin was 96% by normalization of the peak areas detected by HPLC–DAD analysis.

### 2.3. Cell Cultures and Osteoclast Differentiation

This study was carried out in strict accordance with the recommendations contained in the Standard Protocol for Animal Study of Sunchon National University. The protocol was approved by the Sunchon National University Institutional Animal Care and Use Committee (SCNU IACUC; Permit No. SCNU IACUC 2016-06). All efforts were made to minimize suffering. 

All cells were cultured in a 37 °C and 5% CO_2_ incubator, and the medium was changed every 3 days. Bone marrow-derived macrophages (BMMs) were obtained from unfractionated bone marrow cells (BMCs) as follows: BMCs were isolated from the tibia and femur of 5-week-old male ICR mice (*n* = 2: Damool Science, KR) by flushing α-minimum essential medium (α-MEM; Invitrogen Life Technologies, Carlsbad, CA, USA) supplemented with 100 U/mL penicillin/streptomycin (Invitrogen, Carlsbad, CA, USA). Cells were incubated on a petri dish in α-MEM supplemented with 10% fetal bovine serum (FBS; Invitrogen Life Technologies, Carlsbad, CA, USA) and 100 U/mL penicillin/streptomycin (10% α-MEM) with 30 ng/mL of mouse recombinant macrophage colony-stimulating factor (M-CSF; PEPROTECH, Rocky Hill, NJ, USA). After 3 days, cells attached to Petri dishes were obtained as BMMs. BMMs were plated at a density of 1 × 10^4^ cells/well in a 96-well tissue culture plate in 10% α-MEM, and cultured with 10 ng/mL of mouse recombinant RANKL (R&D Systems, Minneapolis, MN, USA) and 30 ng/mL M-CSF for 4 days in the presence or absence of samples.

### 2.4. Cytotoxicity Assay

BMMs were plated in a 96-well tissue culture plate in triplicate at a density of 1 × 10^4^ cells/well in 10% α-MEM, and cultured with 30 ng/mL M-CSF and the samples for 3 days. Cell viability was evaluated by using a CCK-8 kit (Dojindo Molecular Technologies, Kumamoto, Japan) according to the manufacturer’s protocol.

### 2.5. Tartrate-Resistant Acid Phosphatase (TRAP) Staining and Activity Assay

Cultured adherent cells were fixed with 3.7% formalin in PBS for 5 min, permeabilized with 0.1% Triton X-100 for 10 min, and incubated for 10 min with a TRAP-staining solution (Sigma-Aldrich, St. Louis, MO, USA). TRAP positive-multinucleated cells (TRAP^+^-MNCs) containing three or more nuclei were counted as mature osteoclasts. Then, we added 100 μL/well of TRAP buffer (100 mM sodium citrate, 50 mM sodium tartrate, 1 mg/mL p-nitrophenyl, pH 5.0) to the 96-well plate and incubated at 37 °C for 1 h. The reaction mixture was transferred to a new 96-well plate containing 90 μL of 0.1 N NaOH, and the absorbance at 410 nm was measured.

### 2.6. Real-Time PCR

BMMs were plated at 3.5 × 10^4^ cells/well in a six-well tissue culture plate, and cultured with 10 ng/mL of RANKL and 30 ng/mL M-CSF for 0, 1, 2, and 3 days in the presence or absence of betulin. Real-time PCR was performed as described previously [24]. Primers for real-time PCR were designed (Table 1) by using the Primer3 design program [25]. Total RNAs were isolated with a TRIzol reagent (Invitrogen, Carlsbad, CA, USA), and 1 μg of RNAs were reverse-transcribed with the M-MLV cDNA Synthesis kit (Enzynomics, Daejeon, Korea) according to the manufacturer’s protocol. Quantitative PCR was accomplished using TOPreal qPCR 2× PreMIX (Enzynomics, Daejeon, Korea) and Real-Time PCR detection system (Bio-Rad, Hercules, CA, USA). All tests were run in triplicate, and glyceraldehyde-3-phosphate dehydrogenase (GAPDH) was used as an internal standard, and data were analyzed by the 2^−ΔΔ*CT*^ method [26].

### 2.7. Western Blot Analysis

Western blotting was performed as described previously [27]. Cells were washed with phosphate-buffer saline (PBS) and homogenized in lysis buffer (50 mM Tris-HCl, 150 mM NaCl, 5 mM EDTA, 1% Triton X-100, 1 mM sodium fluoride, 1 mM sodium vanadate, and 1% deoxycholate) supplemented with 1 mM phenylmethylsulfonyl fluoride (PMSF; Bio Basic, Amherst, NY, USA). The protein concentration was determined by the DC Protein Assay (Bio-Rad, Hercules, Hercules, CA, USA). Protein extracts were subjected to 10% sodium dodecyl sulfate-polyacrylamide gel electrophoresis (SDS-PAGE) (20 μg per lane) and transferred to a polyvinylidene difluoride (PVDF) membrane (Amersham Biosciences, Piscataway, NJ, USA). After blocking with 5% skim milk, the membranes were incubated overnight at 4 °C with primary antibodies. NFATc1 and actin antibodies were from Santa Cruz Biotechnology (Paso Robles, CA, USA). Antibodies against p38, p-p38, c-Jun N-terminal kinase (JNK), p-JNK, extracellular signal-regulated kinase (ERK), and p-ERK were obtained from Cell Signaling Technology (Danvers, MA, USA). After washing, the membranes were incubated with horseradish peroxidase (HRP)-conjugated secondary antibody (Santa Cruz Biotechnology, Paso Robles, CA, USA) for 2 h at room temperature. The antibody blots were treated with Super-Signal West Pico/Femto Chemiluminescent Substrate (Pierce Chemical, Rockford, IL, USA) and developed by using MicroChemi 4.2 (DNR Bio-imaging System, Neve Yamin, Israel).

### 2.8. Bone Pit Formation Assay

BMMs were plated on an Osteo Assay Plate (24 well plate) at a density of 3 × 10^5^ cells/well and stimulated with 10 ng/mL RANKL and 30 ng/mL M-CSF in the presence of betulin (the indicated concentrations). After 4 days, the cells were removed completely, then the resorption area was observed under a light microscope (magnification, × 50; Leica Microsystems, Wetzlar, Germany), and after that, measured by ImageJ software (NIH, Bethesda, MD, USA). For each sample, three fields of vision were examined.

### 2.9. Statistical Analysis

All quantitative data are presented as the mean ± standard deviation of three replicate experiments. Statistical differences were analyzed by applying Student’s *t*-test. Probability (*p*) values less than 0.05 were considered significant (*p* values * < 0.05, ** < 0.01, and *** < 0.001).

## 3. Results

### 3.1. Betulin Was Isolated from B. platyphylla Barks

**Figure 1:**
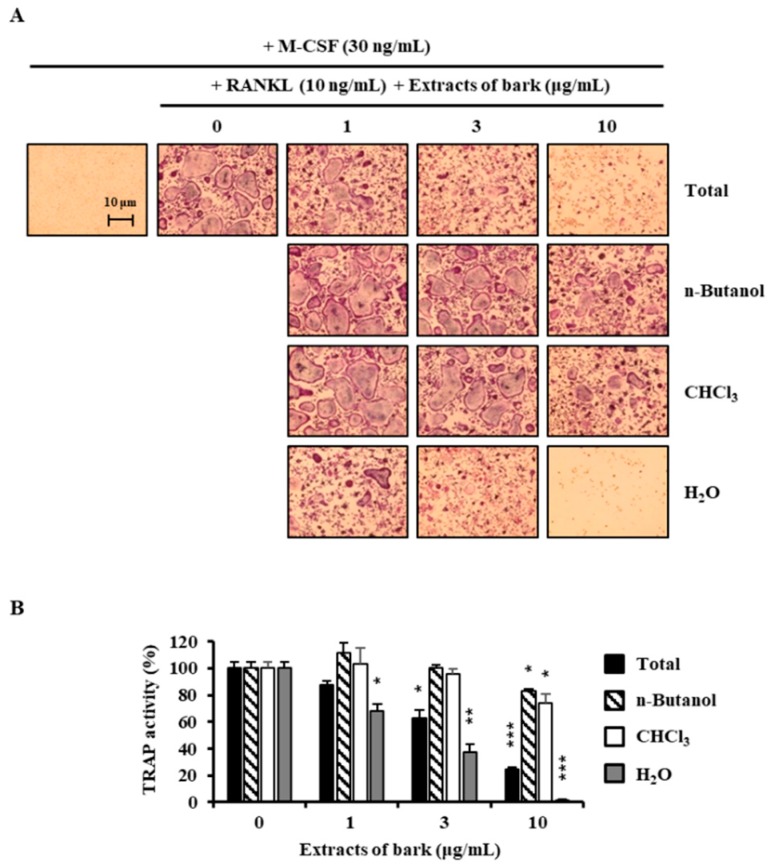
Effects of extracts of *Betula platyphylla* on the differentiation of BMMs into osteoclasts. (**A**) BMMs were cultured for 4 days with RANKL (10 ng/mL) and M-CSF (30 ng/mL) in the presence of the indicated concentrations of extracts. Total: 80% methanol extract of *B. platyphylla* barks, *n*-Butanol: *n*-butanol fractions of the total, CHCl_3_: CHCl_3_ fractions of the total, and H_2_O: H_2_O fractions of the total; (**B**) TRAP activity was measured. *, *p* < 0.05; **, *p* < 0.01; ***, *p* < 0.001 (*n* = 3).

**Figure 2:**
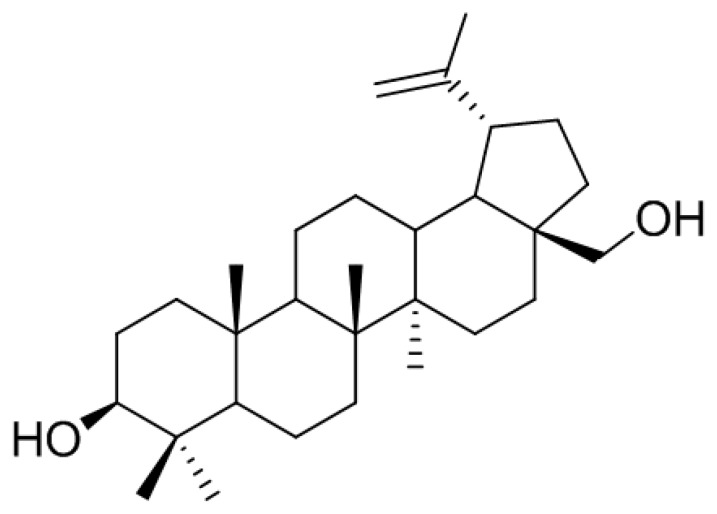
Chemical structure of betulin isolated from *B*. *platyphylla*.

In the search for anti-osteoclastogenic natural products, we found that methanolic extract of *B*. *platyphylla* inner bark displayed a concentration-dependent suppression of the differentiation of BMMs into osteoclasts at the concentrations ranging from 1 μg/mL to 10 μg/mL (Figure 1). Dried and pulverized *B*. *platyphylla* bark was extracted with 80% methanol by using an ultrasonic apparatus. The 80% methanolic extract of *B. platyphylla* bark was suspended in H_2_O and successively fractioned into CHCl_3_, *n*-butanol, and H_2_O fractions. The compound, obtained as a major constituent by recrystallization from *n*-butanol and H_2_O fraction, was isolated as whitish needles. The molecular formula was determined to be C_30_H_50_O_2_ from the negative fast atom bombardment-Mass Spectrometry (FAB-MS) at *m*/*z* = 425 [M-H_2_O+H]^−^. The ^1^H-NMR spectrum in CDCl_3_ showed evidence for the presence of an isopropylene function [δ 4.65 (1H, br s, H-29b), 4.56 (1H, br s, H-29b), 1.66 (3H, s, H-30)], five *tert*-CH_3_ groups [δ 1.05 (3H, s, H-27), 1.03 (3H, s, H-23), 1.02 (3H, s, H-24), 0.99 (3H, s, H-26), 0.96 (3H, s, H-25)], geminal protons [δ 3.77 (1H, br d, *J* = 11.0, H-28a), 3.31 (1H, br d, *J* = 10.6, H-28b)] for the aforesaid lupane type pentacyclic triterpenoid skeleton. The ^1^H-NMR spectrum at δ 3.16 (1H, dd, *J* = 10.8, 5.3 Hz, H-3) and δ 3.77 (1H, br d, *J* = 11.0, H-28a), 3.31 (1H, br d, *J* = 10.6, H-28b) indicated the *β*-orientated hydroxyl group and hydroxymethane group, respectively. The ^13^C-NMR spectrum showed thirty carbon signals. On the basis of the above data, this compound was determined to be betulin [28] (Figure 2).

Betulin. Whitish needles; C_30_H_50_O_2_; Positive FABMS (*m/z*): 425 [M-H_2_O+H]^+^; ^1^H-NMR (400 MHz, CDCl_3_): δ 4.65 (1H, br s, H-29a), 4.56 (1H, br s, H-29b), 3.77 (1H, br d, *J* = 11.0 Hz, H-28b), 3.31 (1H, br d, *J* = 10.6 Hz, H-28a), 3.16 (1H, dd, *J* = 10.8, 5.3 Hz, H-3), 1.00 (3H, s, H-27), 0.95 (3H, s, H-26), 0.94 (3H, s, H-23), 0.80 (3H, s, H-25), 0.73 (3H, s, H-24); ^13^C-NMR (100 MHz, CDCl_3_): δ 150.5 (C-20), 109.7 (C-29), 79.0 (C-3), 60.6 (C-28), 55.3 (C-5), 50.4 (C-9), 48.7 (C-19), 47.8 (C-17,18), 42.7 (C-14), 40.9 (C-8), 38.9 (C-1), 38.7 (C-4), 37.3 (C-10), 37.2 (C-13), 34.2 (C-7), 34.0 (C-22), 29.7 (C-21), 29.2 (C-23), 28.0 (C-2), 27.4 (C-15), 27.0 (C-12), 25.2 (C-11), 20.8 (C-30), 18.3 (C-6), 16.1 (C-25), 16.0 (C-26), 15.3 (C-24), 14.8 (C-27).

### 3.2. Betulin Inhibits the Differentiation of BMMs into Osteoclasts

**Figure 3:**
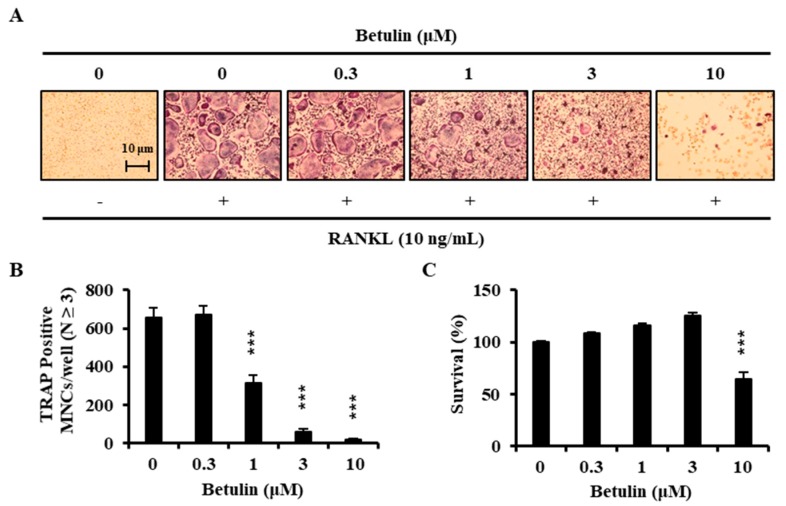
RANKL-induced osteoclast differentiation is inhibited by betulin. (**A**) BMMs were cultured for 4 days with RANKL (10 ng/mL) and M-CSF (30 ng/mL) in the presence of the indicated concentrations of botulin; (**B**) Multinucleated cells were fixed (3.7% formalin), permeabilized (0.1% Triton X-100), and stained with TRAP solution. Mature TRAP^+^-MNCs (nuclei ≥ 3) were photographed under a light microscope. TRAP-positive MNCs (nuclear number > 3) were counted. ***, *p* < 0.001 (*n* = 3); (**C**) The effect of betulin on the viability of BMMs was evaluated by the CCK-8 assay. ***, *p* < 0.001 (*n* = 3).

To examine the effects of betulin on the RANKL-induced osteoclast differentiation, BMMs were incubated with 30 ng/mL M-CSF and 10 ng/mL RANKL in the presence of betulin or vehicle (0.1% DMSO) for 4 days. Figure 3A shows that RANKL significantly induced TRAP^+^-osteoclast differentiation. However, the betulin above 3 μM considerably inhibited the formation of TRAP^+^-osteoclasts. Consistent with these results, the number of TRAP^+^-MNCs (nuclei ≥ 3) was decreased by betulin in a dose-dependent manner (Figure 3B).

### 3.3. Betulin Had No Cytotoxic Effect

We confirmed the toxic effect of betulin on the BMMs to determine whether betulin inhibited osteoclast differentiation through toxicity. BMMs were incubated with betulin in the presence of 30 ng/mL M-CSF for 3 days. Betulin showed significant cytotoxicity above 10 μM, whereas there was no cytotoxic effect under 3 μM (Figure 3C). These results implied that betulin had the inhibitive activity against the differentiation of osteoclast without any cytotoxicity.

### 3.4. Betulin Suppressed RANKL-Induced Expression of NFATc1

**Figure 4:**
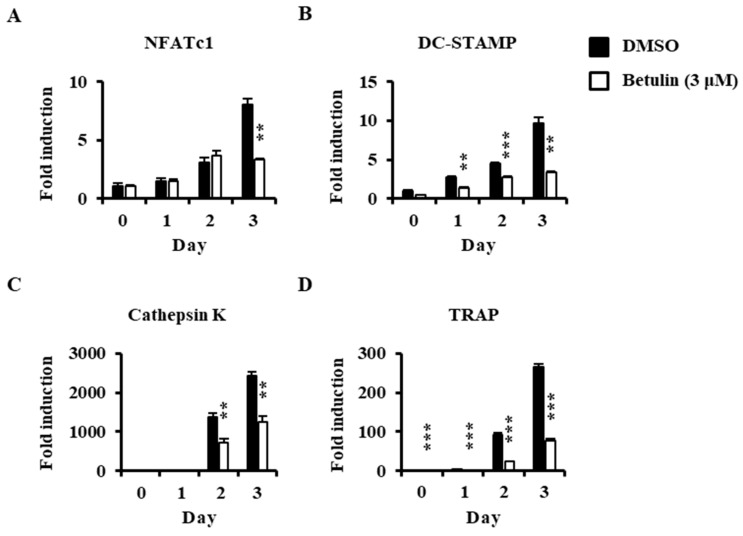
Effect of betulin on RANKL-induced mRNA expressions of osteoclastic-specific genes. BMMs were treated with vehicle (0.1% DMSO) or betulin (3 μM) for 1 h, and then RANKL (10 ng/mL) and M-CSF (30 ng/mL) were treated for the indicated time periods. Total RNA was then isolated using a TRIzol reagent, and mRNA expression levels were evaluated by real-time PCR. The TRIzol reagents were used: (**A**) NFATc1; (**B**) DC-STAMP; (**C**) Cathepsin K; (**D**) TRAP. Glyceraldehyde-3-phosphate dehydrogenase (GAPDH) was used as the internal control. **, *p* < 0.01; ***, *p* < 0.001 (*n* = 3).

The anti-osteoclastogenic activity of betulin on the expression of transcription factors and osteoclast-specific markers were confirmed by real-time PCR analysis. Betulin inhibited the mRNA expression of NFATc1 (transcription factors in osteoclastogenesis) in response to RANKL (Figure 4A). Furthermore, the mRNA levels of NFATc1-related molecules, such as DC-STAMP, cathepsin K, and TRAP, were also significantly suppressed by betulin in osteoclast differentiation (Figure 4B–D).

**Figure 5:**
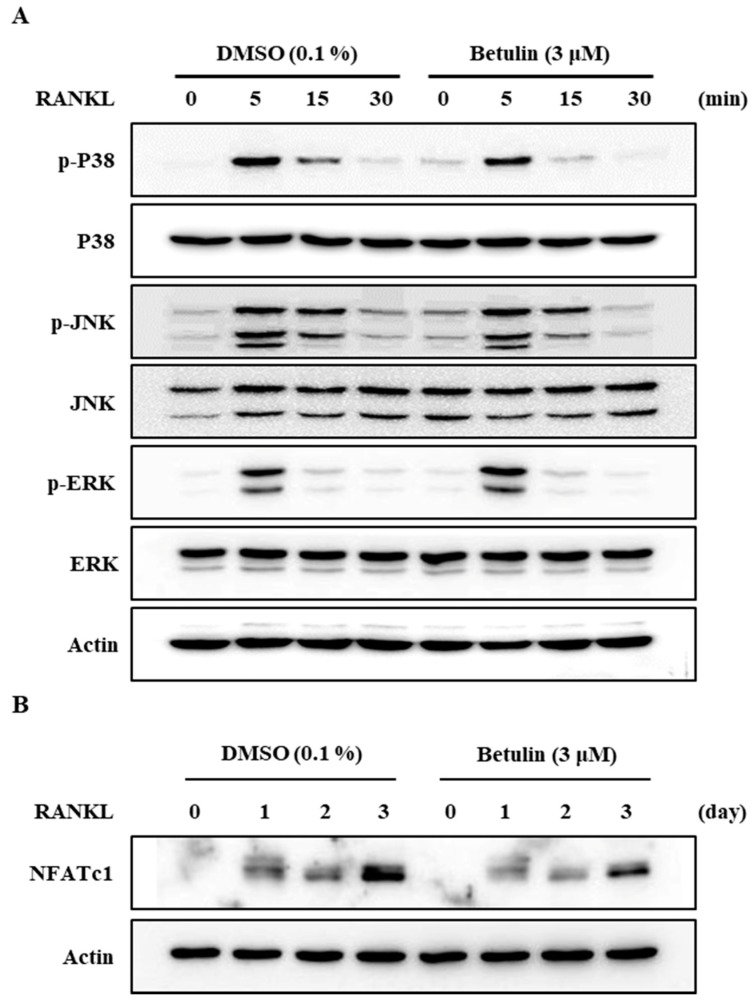
Betulin downregulated RANKL-mediated activation of NFATc1 through inhibition of p38 phosphorylation in BMMs. The effects of betulin on RANKL-induced phosphorylation of MAP kinases and degradation of I-kB and NFATc1 were evaluated by Western blot analysis. Actin was used as an internal control. (**A**) BMMs were pretreated with vehicle (0.1% DMSO) or betulin (3 μM) for 1 h prior to RANKL (10 ng/mL) stimulation at the indicated time periods; (**B**) BMMs were treated with vehicle (0.1% DMSO) or betulin (3 μM) for 1 h, and then RANKL (10 ng/mL) and M-CSF (30 ng/mL) were treated at the indicated time periods.

Additionally, to confirm this finding, we analyzed the protein expression level of NFATc1, a master regulator of osteoclast differentiation, by Western blotting. RANKL significantly increased the protein expression of NFATc1, but betulin dramatically inhibited the NFATc1 protein expression level in osteoclast differentiation (Figure 5B). This result confirmed that betulin decreased protein expression of NFATc1, and then the differentiation of osteoclast was inhibited during osteoclastogenesis.

### 3.5. Betulin Inhibited RANKL-Induced Activation of p38

It is well known that the activity of a mitogen-activated protein kinase (MAPK) is important for osteoclast formation [7,10]. We, therefore, assessed the phosphorylation of p38, JNK, and ERK after RANKL stimulation in BMMs using Western blot analysis. The results demonstrated that betulin reduced the RANKL-mediated phosphorylation of p38 and did not affect the others (Figure 5A).

### 3.6. Effects of Betulin on Bone Resorptive Activity in RANKL-Induced Osteoclasts

**Figure 6:**
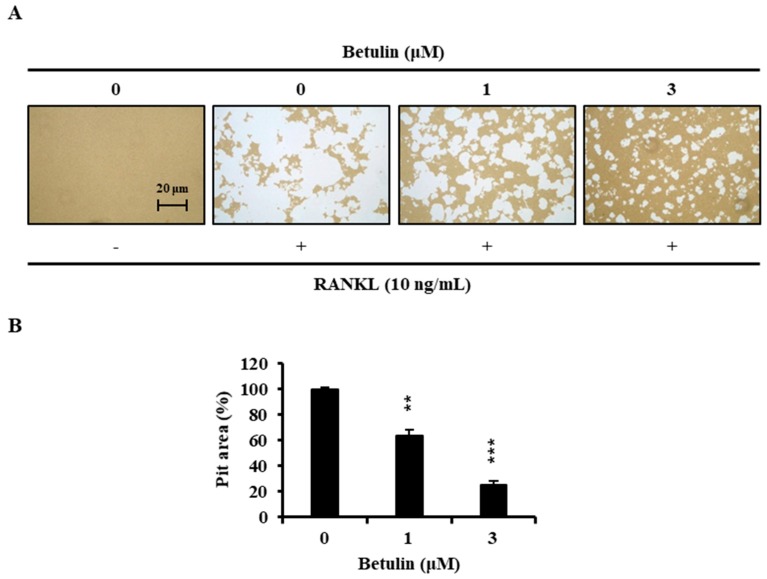
Betulin inhibited RANKL-induced bone resorption by osteoclasts. (**A**) BMMs were plated on an Osteo Assay Plate and treated with 10 ng/mL RANKL and 30 ng/mL M-CSF in the presence of different concentrations of betulin. Following 4 days of culture, the attached cells on the Osteo Assay Plate were removed and photographed under a light microscope; (**B**) Pit areas were quantified using the ImageJ program. **, *p* < 0.01; ***, *p* < 0.001 (*n* = 3).

Finally, we examined whether osteoclast formation inhibited by betulin also affected bone resorption. Wide resorption pits were formed by osteoclasts on the bone slices, but betulin decreased the area of the bone resorption pits in a dose-dependent manner (Figure 6).

## 4. Discussion

Betulin is a naturally occurring triterpene that is often found in birch bark and is also found in the sap. Betulin is an interesting precursor with potentially important biological activity. Betulinic derivatives, such as betulinic acid, have been reported to have anti-tumor, anti-diabetic, anti-malarial, anti-inflammatory, and antifungal activity [21,29,30]. In addition, betulinic acid has been reported to inhibit osteoclast differentiation [22,23]. However, there is no study examining betulin in osteoclasts. Here, we examined the potential for the anti-osteoclastogenesis of betulin.

The osteoclast, which exists only in the bone, is the only cell that resorbs the bone. Therefore, it plays an important role in the sustaining of bone mass or bone strength and is a major cause of bone disease-related bone loss, including osteoporosis [31]. M-CSF and RANKL are important for osteoclast formation. M-CSF plays an important role in the proliferation and survival of macrophage progenitor cells, as well as osteoclasts, and it also stimulates RANK expression in monocytes/macrophage progenitor cells, allowing them to efficiently respond to RANKL [13]. The binding of RANKL and RANK in macrophages promotes the expression of transcription factor NFATc1 [32,33]. NFATc1 is a key regulator in osteoclast differentiation and upregulates TRAP, DC-STAMP, and cathepsin K, which are osteoclast differentiation markers. 

Here, we investigated the effect of betulin on osteoclast differentiation. At first, before obtaining pure betulin from *B. platyphylla* bark, their ability of crude extracts to inhibit osteoclast differentiation was determined using an 80% methanol extract of *B. platyphylla* bark and its CHCl_3_, *n*-butanol, and H_2_O fractions. RANKL-mediated osteoclast differentiation was greatly inhibited by the total methanol extracts of *B. platyphylla* bark. The total methanol extract was fractionated n-butanol, chloroform, and water, where water extracts showed the greatest inhibitory effect on osteoclast differentiation. We crystallized the main component in *n*-butanol and water fractions, and then we confirmed it as betulin by NMR spectroscopic data. Betulin showed similar results to the osteoclast differentiation experiment with the extract from *B. platyphylla* bark, and inhibited the formation of osteoclasts at concentrations above 3 μM. Betulin showed cytotoxicity at concentrations above 10 μM. These results indicated that betulin at 3 μM or less had anti-osteoclastogenic activity without cytotoxicity in BMM. It was confirmed that betulin reduced osteoclast formation. We examined the gene expression of NFATc1, the most important factor in osteoclastogenesis. The mRNA expression level of NFATc1 was inhibited by betulin, and the mRNA expression level of the specific gene in osteoclast differentiation, such as DC-STAMP, cathepsin K, and TRAP, was also significantly decreased by betulin. In addition, we investigated the effect of betulin on the MAPK signaling pathways because activation of the MAPK signaling pathways is important for RANKL-mediated osteoclast differentiation [10]. Betulin inhibited the activation of p38 by RANKL, but did not affect the activity of JNK and ERK. In addition, the protein expression level of NFATc1 was also reduced by betulin, such as gene expression levels. These results suggest that betulin inhibits osteoclastogenesis by decreasing the expression of NFATc1, including inhibition of p38 signaling in RANKL-mediated osteoclast differentiation. Furthermore, we confirmed that bone resorption was inhibited by betulin. Our results suggested that betulin had the potential for the treatment of bone disease, and might be used as a new structural scaffold for osteoclast differentiation inhibitors.

## 5. Conclusions

Betulin inhibited osteoclastogenesis by blocking the expression of NFATc1. Reduced NFATc1 subsequently affected osteoclast differentiation by decreasing osteoclast-related factors, such as DC-STAMP, cathepsin K, and TRAP. Regarding the clinical aspect, betulin might be beneficial in the treatment of bone diseases, such as osteoporosis and rheumatoid arthritis.

## Figures and Tables

**Table 1:** Primer sequences used in this study.

Gene of Interest	Primer Sequence (5′→3′)	
	Sense	Anti-Sense
*NFATc1*	GATGACTTTGCCAGTCAGCA	ACATAGCCCACACCGTTCTC
*GAPDH*	AACTTTGGCATTGTGGAAGG	ACACATTGGGGGTAGGAACA
*Cathepsin K*	GATGACTTTGCCAGTCAGCA	ACATAGCCCACACCGTTCTC
*DC-STAMP*	CCAAGGAGTCGTCCATGATT	GGCTGCTTTGATCGTTTCTC
*TRAP*	GATGACTTTGCCAGTCAGCA	ACATAGCCCACACCGTTCTC
